# Limited Interactions between Streptococcus Suis and Haemophilus Parasuis in In Vitro Co-Infection Studies

**DOI:** 10.3390/pathogens7010007

**Published:** 2018-01-06

**Authors:** Annabelle Mathieu-Denoncourt, Corinne Letendre, Jean-Philippe Auger, Mariela Segura, Virginia Aragon, Sonia Lacouture, Marcelo Gottschalk

**Affiliations:** 1 Swine and Poultry Infectious Diseases Research Center (CRIPA) and Groupe de Recherche sur les Maladies Infectieuses en Production Animale, Department of Pathology and Microbiology, Faculty of Veterinary Medicine, University of Montreal, 3200 Sicotte St., Saint-Hyacinthe, QC J2S 2M2, Canada; annabelle.mathieu-denoncourt@umontreal.ca (A.M.-D.); corinne.letendre@umontreal.ca (C.L.); jean-philippe.auger.1@umontreal.ca (J.-P.A.); mariela.segura@umontreal.ca (M.S.); sonia.lacouture@umontreal.ca (S.L.); 2 IRTA, Centre de Recerca en Sanitat Animal (CReSA, IRTA-UAB), Campus de la Universitat Autonoma de Barcelona, 08193 Bellaterra, Barcelona, Spain; virginia.aragon@irta.cat

**Keywords:** *Streptococcus suis* serotype 2, *Haemophilus parasuis*, co-infections, in vitro, inflammation, phagocytosis, alveolar macrophages, tracheal epithelial cells

## Abstract

*Streptococcus suis* and *Haemophilus parasuis* are normal inhabitants of the porcine upper respiratory tract but are also among the most frequent causes of disease in weaned piglets worldwide, causing inflammatory diseases such as septicemia, meningitis and pneumonia. Using an in vitro model of infection with tracheal epithelial cells or primary alveolar macrophages (PAMs), it was possible to determine the interaction between *S. suis* serotype 2 and *H. parasuis* strains with different level of virulence. Within *H. parasuis* strains, the low-virulence F9 strain showed higher adhesion levels to respiratory epithelial cells and greater association levels to PAMs than the high-virulence Nagasaki strain. Accordingly, the low-virulence F9 strain induced, in general, higher levels of pro-inflammatory cytokines than the virulent Nagasaki strain from both cell types. In general, *S. suis* adhesion levels to respiratory epithelial cells were similar to *H. parasuis* Nagasaki strain. Yet, *S. suis* strains induced a significantly lower level of pro-inflammatory cytokine expression from epithelial cells and PAMs than those observed with both *H. parasuis* strains. Finally, this study has shown that, overall and under the conditions used in the present study, *S. suis* and *H. parasuis* have limited in vitro interactions between them and use probably different host receptors, regardless to their level of virulence.

## 1. Introduction

*Streptococcus suis* (*S. suis*) and *Haemophilus parasuis* (*H. parasuis*) are among the most frequent causes of disease in weaned piglets worldwide [1]. Both bacterial species, mainly low virulent strains, are also normal inhabitants of the porcine upper respiratory tract and are present in most healthy animals [2,3]. They are both transmitted by nasal contact from a colonized animal to another, usually from the sow to the piglets but also among piglets [2,3]. Both pathogens cause inflammatory infections such as septicemia, polyserositis, meningitis, arthritis and pneumonia [2,3]. 

*S. suis* is classified into 35 serotypes, based on the antigenicity of the capsular polysaccharide (CPS). More recently, some serotypes (20, 22, 26, 32, 33 and 34) have been suggested to belong to different bacterial species [4], whereas strains with new capsular genes have also been described [5]. Serotype 2 has been described as being the most virulent and frequently recovered serotype from diseased animals [6]. However, phenotypic and genotypic differences within serotype 2 strains do exist [7]. The use of multilocus sequence typing has revealed that some strains of serotype 2 belonging to certain sequence types (STs) are more virulent than others. For example, virulent ST1 (as well as other members of the clonal complex 1) strains predominate in most Eurasian countries, whereas ST25 and ST28 strains (intermediate and low virulence, respectively) are widely distributed in North America [7]. The early steps of a *S. suis* infection take place in the upper respiratory tract. Bacteria adhere and, to a certain extent, invade the epithelial cells [8]. Although mechanisms are not completely understood, *S. suis* eventually reaches the bloodstream, remains extracellular by resisting phagocytosis and causes disease [3]. *S. suis* resistance to phagocytosis by phagocytic cells is mainly due to the presence of the CPS [9], which may indeed affect not only its own phagocytosis but also that of an heterologous species, such as Group B *Streptococcus* [10]. Bacteria then induce the production of pro-inflammatory cytokines in the respiratory tract as well as systemically that may compromise the host [11]. 

*H. parasuis* is the etiological agent of Glässer’s disease and is classified into 15 serotypes [12]. There are virulent and low-virulent strains and there is no clear relationship between virulence and serotype [2]. Many of the non-typeable strains identified as such by the use of antibodies can now be serotyped by PCR [13,14]. Virulent strains of *H. parasuis* are able to colonize and initiate infection by adhesion to and, to a certain extent, invasion of epithelial cells [15]. In the lungs, non-virulent strains of *H. parasuis* can be eliminated through phagocytosis by alveolar macrophages [16]. In contrast, virulent strains of *H. parasuis* are able to avoid phagocytosis, probably due, among other factors, to the expression of a bacterial capsule, which allows multiplication of bacteria inside the host with a production of a strong inflammatory reaction that results in the characteristic lesions of Glässer’s disease [2]. Once virulent strains enter the bloodstream (by still unknown mechanisms), the bacterium is able to avoid complement-mediated killing in an antibody-independent manner [2]. *H. parasuis* is also able to cause bronchopneumonia and virulence of isolates recovered from affected lungs is not completely known, since these isolates may also be the result of aspiration of low virulent colonizers from the upper respiratory tract [17]. Different virulence factors have been suggested to play important roles in the pathogenesis of the Glässer’s disease [2]. Among them, the virulence associated trimeric autotransporters (VtaA) are those more characterized and their presence has been used to potentially identify virulent isolates by PCR [18].

Because *S. suis* and *H. parasuis* are present in the upper respiratory tract and both cause inflammatory diseases in young piglets after weaning, interactions between the two species may occur during the early steps of infection. In the present study, the interactions of virulent and intermediate/low-virulent *S. suis* and *H. parasuis* strains with Newborn Pig Tracheal cells (NPTr) and primary porcine alveolar macrophages (PAMs) during single infections and as well as simultaneous or sequential co-infections were studied. Results indicate that, in general, limited interaction occurs between the two bacterial species.

## 2. Results and Discussion 

### 2.1. Co- or Sequential Infections of Swine Tracheal Epithelial Cells by S. suis and H. parasuis Has No Impact on Their Adhesion/Invasion Capacities

Both *H. parasuis*, as *S. suis*, first colonize pigs in their upper respiratory tract [2], through adhesion and, to some extent, invasion of epithelial cells [15]. To determine the effect of a co-infection on the early steps of infection in swine, the adhesion to and the invasion of swine epithelial cells from upper respiratory tract (NPTr) by strains of *S. suis* and *H. parasuis* of different virulence was evaluated. For the first time, individual cell adhesion of both bacterial species was compared within the same study and under the same conditions. All studies have been done under non-toxic conditions (<15%), as revealed by the lactate dehydrogenase (LDH) test (results not shown). 

**Figure 1:**
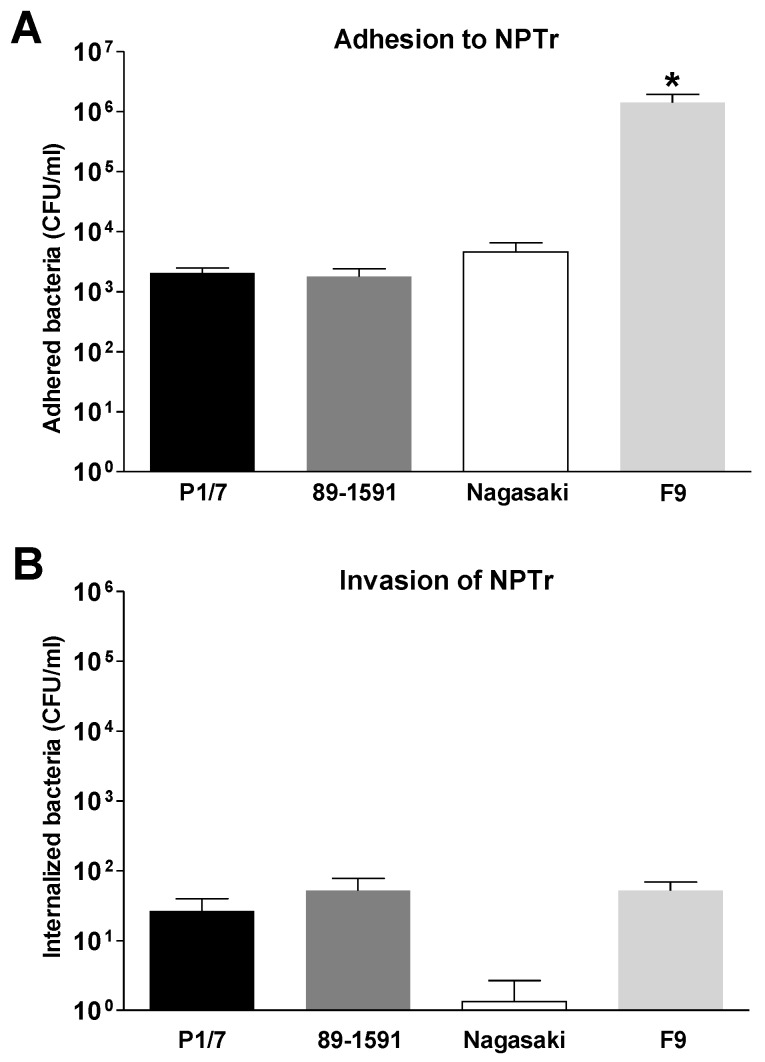
Adhesion to and invasion of Newborn Pig Tracheal cells (NPTr) epithelial cells by *S. suis* or *H. parasuis* strains of different virulence. NPTr cells were infected with *S. suis* serotype 2 strains P1/7 (high virulence) or 89-1591 (intermediate virulence), or with *H. parasuis* strains Nagasaki (high virulence) or F9 (low virulence) with a MOI of 5 for 2 h. (**A**) After the incubation time, cells were washed to remove non-adherent bacteria and lyzed in pure water to determine the number of adherent bacteria per well. (**B**) Infected cells were washed and cell culture media was replaced by fresh media with antibiotics and further incubated for 2 h in order to kill extracellular bacteria. Cells were washed to remove the antibiotics and lyzed in pure cold water to release internalized bacteria. Levels of adhesion/invasion are expressed as the total number of colony forming units (CFU) recovered per well. Data are expressed as means ± standard errors from at least four independent experiments. An asterisk indicates significant differences between samples (*p* < 0.05).

As shown in Figure 1A, after 2 h of single infection and an MOI of 5, both high virulent and intermediate virulent *S. suis* serotype 2 strains presented similar level of adhesion to swine tracheal epithelial cells. Similar results were observed when using different MOIs and incubation times (results not shown). Obtained results were similar to what has previously been described for this pathogen [19]. Most *S. suis* adhesion studies previously done used the low relevant human epithelial cell line HEp-2 [9]. In fact, a few studies have been carried out with swine tracheal epithelial cells and *S. suis*, all of them also showing similar adhesion levels to those observed in the present study [11,19,20,21]. Interestingly, the intermediate virulent North American ST25 strain 89-1591 presented similar adhesion levels than the high virulent ST1 strain P1/7 (Figure 1A). Previous studies with *S. suis* serotype 2 have exclusively used virulent ST1 strains, which produce a haemolysin, called suilysin [22]. In fact, suilysin was shown to have a positive effect on *S. suis* adhesion to epithelial cells in absence of CPS [23]. However, the ST25 North-American strain used in this study (as well as all ST25 strains described so far) does not produce the suilysin, indicating that in the presence of the CPS this haemolysin might not be a critical factor for adhesion. Similar very low invasion rates have been obtained for both strains (Figure 1B), which confirm a previous study using an ST1 strain and the same cells [19]. This may suggest that high and intermediate virulent strains do not differ in these first steps of the pathogenesis of *S. suis* and differences may probably take place during the systemic phase. 

The virulent *H. parasuis* Nagasaki strain adhered to epithelial cells in similar levels than both *S. suis* strains (Figure 1A). However, the low-virulent *H. parasuis* strain F9 adhered in significantly higher levels when compared to all other strains (*p* < 0.05) (Figure 1A). Similar results were observed when using different MOIs and incubation times (results not shown). Adherence of Nagasaki strain to epithelial cells has been previously shown [15]. Interestingly, in that study, authors showed higher levels of adhesion for this strain to renal epithelial cells, when compared to the low-virulent strain SW114 [15]. These results differ from those obtained in the present study; it is difficult to establish if differences in adhesion between SW114 and F9 strains are really due to their virulence potential or to the cell type (tracheal vs. renal) used in the adhesion test and further studies are needed to obtain a definitive conclusion. Two previous studies also showed similar low levels of adhesion with Nagasaki strain and NPTr cells [24,25]. In the present study, both strains of *H. parasuis* presented very low levels of invasion (less than 1 bacteria internalized per 2000 cells) (Figure 1B) and no significant differences were observed between the strains (*p* = 0.181). Invasion of *H. parasuis* to epithelial cells is still controversial and, as it is the case for adhesion, there are important differences between results obtained from renal and respiratory epithelial cells [15,24,25].

**Figure 2:**
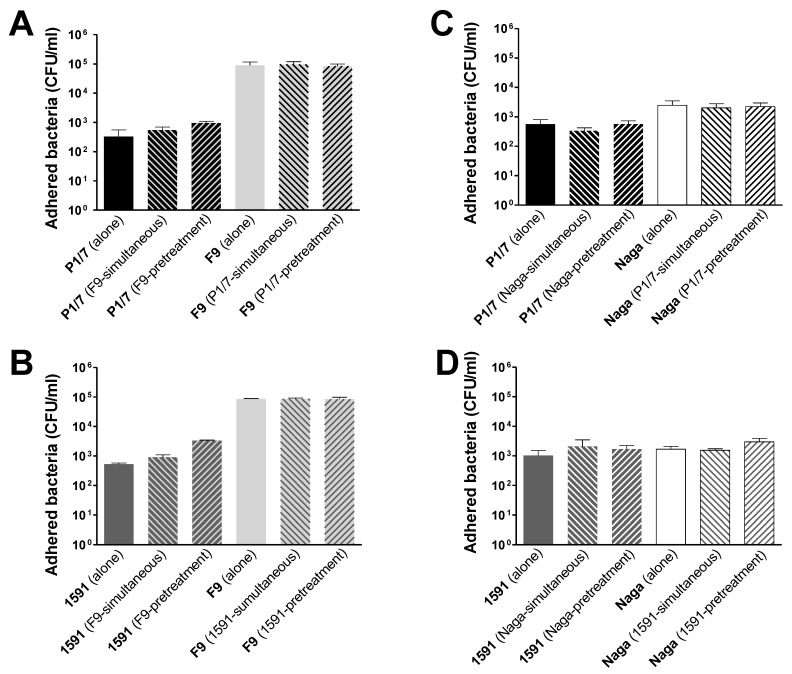
Adhesion of *S. suis* and *H. parasuis* strains of different virulence to Newborn Pig Tracheal cells (NPTr) epithelial cells either alone or in simultaneous or sequential co-infections. NPTr cells were pre-infected with *S. suis* or with *H. parasuis* strains (MOI of 5) for 15 min. Cells were then washed and infected with *H. parasuis* or *S. suis* strains, respectively (MOI of 5) and further incubated for 2 h. Separate wells were also kept with cell culture media (mock infected) as controls. In parallel experiments, cells were infected with both bacterial species simultaneously and also incubated for 2 h. Cells were washed to remove non-adherent bacteria and lyzed in pure water to liberate adherent bacteria. Levels of adhesion are expressed as the total number of colony forming units (CFU) recovered per well. Full bars match single infections. Hatched bars match co-infections. (**A**) Adhesion of high-virulent P1/7 and low-virulent F9 in co-infection. (**B**) Adhesion of intermediate-virulent 89-1591 and low-virulent F9 in co-infection. (**C**) Adhesion of high-virulence P1/7 and high-virulent Nagasaki (Naga) during co-infection. (**D**) Adhesion of intermediate virulent 89-1591 and high-virulent Nagasaki in co-infection. Data are expressed as means ± standard errors from at least four independent experiments.

When the two bacterial species were used simultaneously, no differences in the adhesion of *S. suis* or *H. parasuis* could be detected when compared to those observed with individual infections (Figure 2). Similarly, a pre-infection with *S. suis* did not have any effect on *H. parasuis* adhesion to cells and vice-versa (Figure 2). All results were identical independently of the virulence of the strain. Even the use of a first bacterial species at an MOI: 500 to attempt to block cell receptors during the pre-infection did not influence the adhesion of the second bacterial species (results not shown). Similarly, it has not been possible to observe any significant difference in the bacterial invasion of the tracheal cells during co-infections or pre-infections compared to single infections (results not shown). All these results indicate that these bacterial species probably use different cell receptors. Knowledge on epithelial cell host receptors recognized by *S. suis* is limited [8]. A sialic acid-rich carbohydrate receptor (NeuNAca2-3Galb1-4GlcNAcb1-3Gal), host cell surface glycosaminoglycans and the globotriaosylceramide (GbO3) have all been suggested as possible receptors for epithelial cells [8]. On the other hand, receptors involved in *H. parasuis* adhesion/invasion of epithelial cells are unknown. 

### 2.2. H. parasuis Induces a Higher Expression of Pro-Inflammatory Cytokines by Tracheal Porcine Epithelial Cells and Primary Alveolar Macrophages than S. suis and Co-Infections Only Partially Modulate This Expression 

Inflammation seems to be a hallmark of *S. suis* infections [3]. Although it is not a typical respiratory pathogen, *S. suis* may complicate infections caused by other aetiological agents of the Porcine Respiratory Diseased Complex, such as influenza [19]. It is generally accepted that one of the main role of *S. suis* would be to increase local inflammation [11]. As such, *S. suis* is able to induce inflammatory mediators from respiratory epithelial cells [11,19]. Interestingly, only one study addressed the inflammatory response of PAMs by *S. suis* [26]. Remarkably, the latter as well as all other studies of *S. suis* serotype 2 and swine cells have all been performed with virulent ST1 strains and almost no information is available for North American strains belonging to other STs of lower virulence. 

On the other hand, it has also been described that inflammation is an important player in the pathogenesis of the Glässer’s disease [2]. It has been previously shown that epithelial cells may highly contribute to local inflammation observed with this pathogen [24]. In addition, if *H. parasuis* reaches the lungs, virulent strains resist to phagocytosis by PAMs and delay the activation of those cells, leading to bacterial multiplication in the lung and, ultimately, the release of inflammatory mediators resulting in pneumonia and polyserositis [16,27]. For unknown circumstances, strains with a low-virulent profile (based on autotransporters analysis) are, in some cases, isolated from the lungs of pigs with pneumonia, which may indicate they are poorly phagocytosed [18]. *H. parasuis* would then induce the production of IL-6 and IL-8 and the apoptosis of respiratory epithelial cells, which may lead to its entry into the bloodstream [24,28]. 

**Figure 3:**
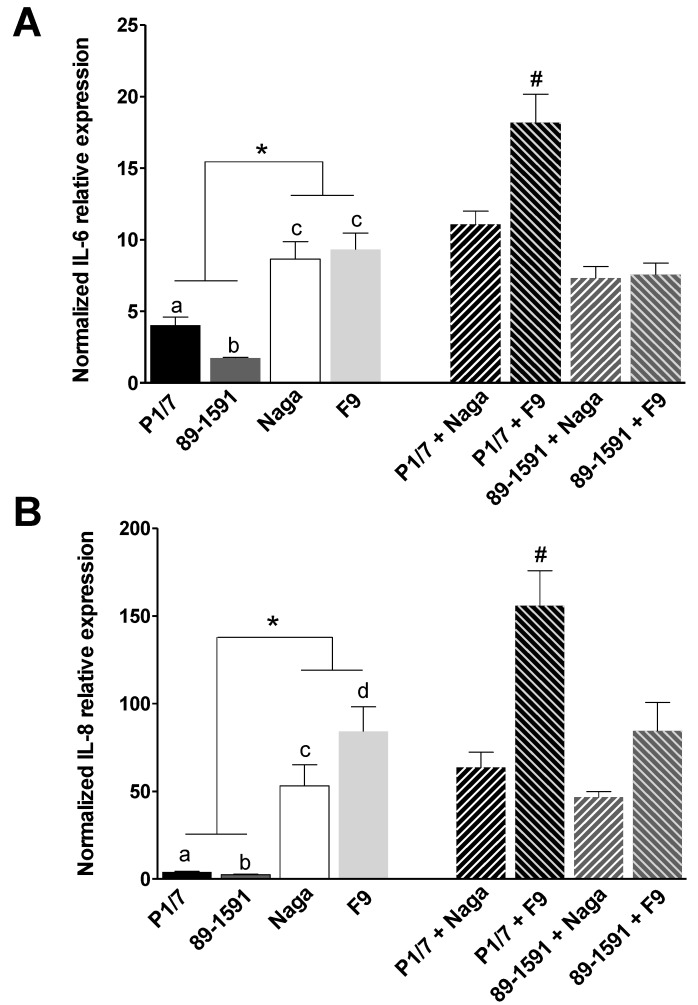
Normalized relative expression of IL-6 (**A**) and IL-8 (**B**) by Newborn Pig Tracheal cells (NPTr) epithelial cells during single and simultaneous co-infections with *S. suis* and *H. parasuis*. Cells were infected with *S. suis* (high-virulent P1/7 or intermediate virulent 89-1591 strains) or with *H. parasuis* (low-virulent F9 or high-virulent Nagasaki [Naga] strains) alone or in simultaneous co-infections for 6 h. Gene expression levels were analysed by RT-qPCR and normalized with the expression of PPIA and Hypox. Relative fold differences were calculated compared to non-infected cells. Data represent mean values ± standard errors of the mean from at least four independent experiments. Full bars show results from single infections. Hatched bars show results from simultaneous co-infections. Different letters indicate significant differences between two strains of the same bacterial species (*p* < 0.05). *, indicates significant differences between *S. suis* and *H. parasuis* strains (*p* < 0.05). #, indicates significant synergistic effect on cytokine production when compared respective single infections (*p* < 0.05).

**Figure 4:**
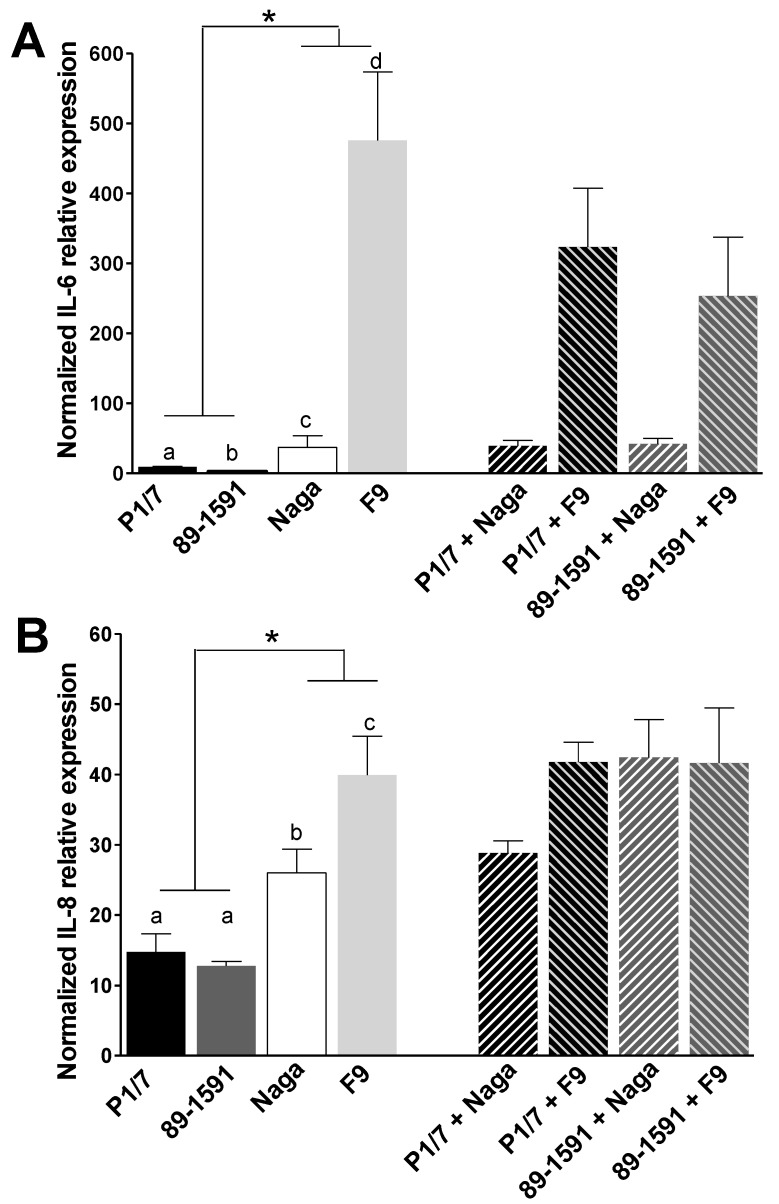
Normalized IL-6 (**A**) and IL-8 (**B**) relative expression by primary alveolar macrophages (PAMs) after single and simultaneous co-infections with *S. suis* and *H. parasuis*. PAMs were infected with either *S. suis* (high and intermediate virulent P1/7 and 89-1591 strains, respectively) or with *H. parasuis* (high and low virulent Nagasaki [Naga] and F9 strains, respectively) alone or in simultaneous co-infection for 12 h. Expression of genes was analysed by RT-qPCR and normalized with the expression of PPIA and Hypox genes. Relative fold differences were calculated compared to the non-infected cells. Data represent mean values ± standard errors of the mean from at least four independent experiments. Full bars show results from single infections. Hatched bars show results from simultaneous co-infections. Different letters indicate significant differences between two strains of the same bacterial species (*p* < 0.05). *, indicates significant differences between *S. suis* and *H. parasuis* strains (*p* < 0.05).

In the present study, we aimed to compare the inflammatory response of respiratory epithelial cells and PAMs infected with strains of *H. parasuis* and/or *S. suis* of different virulence. As it was the case with epithelial cells, PAM studies were done under non-toxic conditions (results not shown). First, swine tracheal epithelial cells and PAMs were infected with *S. suis* or *H. parasuis* alone in order to compare the expression of pro-inflammatory cytokines induced by each species and according to the virulence of the strains (Figure 3 and Figure 4, respectively). Cells were infected for 6 h (NPTr) or 12 h (PAMs) and a quantitative RT-PCR was performed to assess the relative expression of IL-6 and IL-8, normalized by using Peptidylprolyl isomerase A (PPIA) and hypoxanthine phosphoribosyl transferase 1 (Hypox) genes and compared to the expression in non-infected cells. These incubation times were shown to be optimal for gene expression under non-toxic conditions during preliminary studies (results not shown). In general, *H. parasuis*, whether it is the virulent or the low-virulent strain, induced a higher expression of IL-6 and IL-8 by the epithelial cells during single infections than *S. suis*. Indeed, the latter induced a limited expression of both cytokines (Figure 3). *S. suis* and *H. parasuis* have been described to activate cells through Toll-like receptors (TLR) [29,30], some of them (such as TLR2 and 6) being common to both species. However, the role of each receptor is probably different, being *H. parasuis* and *S. suis* a Gram-negative and a Gram–positive microorganism, respectively. Only two studies are available in the literature concerning cytokine expression by epithelial cells infected with *H. parasuis* [24,31]. Although some differences were observed in one of these studies when field strains of serotypes 4 and 5 were compared [24], this does not seem to be related to the virulence of the strain. In this study, the low-virulent F9 strain induced higher levels of IL-8 expression than the virulent strain, which might be related to the higher capacity of this strain to adhere to cells. Additional strains should be tested to reach a definitive conclusion. In the case of *S. suis*, expression of both cytokines with high-virulent strains has been previously shown with these cells [11]. Interestingly, the high-virulent P1/7 strain induced a higher expression of IL-6 (4.03 ± 1.28) and IL-8 (4.02 ± 0.80) than the intermediate virulent 89-1591 strain (1.75 ± 0.99 and 2.44 ± 0.63, respectively) (Figure 3). Whether or not the capacity to stimulate epithelial cells by strains of different virulence has an influence on the pathogenesis of the infection remains to be confirmed.

When epithelial cells were co-infected with the highly virulent ST1 *S. suis* P1/7 and *H. parasuis* Nagasaki strains, only an additive effect on the expression of IL-6 and IL-8 was observed (Figure 3). However, a synergic effect (*p* < 0.05) on the expression of both cytokines could be observed with a co-infection of the low-virulent strain F9 and the highly virulent strain P1/7 (Figure 3). These results might indicate that a virulent strain of *S. suis* may positively modulate the expression of pro-inflammatory cytokines in the presence of a low-virulent *H. parasuis* strain, which might increase the local virulence in the presence of both bacterial species. However, more strains of known virulence should be tested before this hypothesis is confirmed.

In infected PAMs, *H. parasuis* strains also induced higher levels of IL-6 and IL-8 mRNA than both *S. suis* strains (Figure 4). In addition, the low-virulent F9 strain induced statistically higher levels of IL-6 (Figure 4A) and IL-8 (Figure 4B) than the virulent strain Nagasaki, probably due to higher levels of bacterial association to cells (see below). As observed with epithelial cells, *S. suis* high-virulent P1/7 strain induced higher IL-6 RNA levels (Figure 4A) than the intermediate-virulence 89-1591 strain. When analyzing co-infections, neither a clear additive nor a synergistic effect on the expression of IL-6 was observed when the PAMs were infected with *S. suis* and *H. parasuis* strains (Figure 4A). An additive effect on the expression levels of IL-8 by PAMs co-infected with *S. suis* intermediate virulent strain (89-1591) and virulent *H. parasuis* Nagasaki strain was observed (Figure 4B). Other strain combinations did not present any additive or synergistic effect. Overall, these results differ from those observed with co-infected epithelial cells. Indeed, no explanation for such specific interaction with those strains and PAMs could yet be found.

### 2.3. S. suis Has No Effect on H. parasuis Association to or Phagocytosis by PAMs 

**Figure 5:**
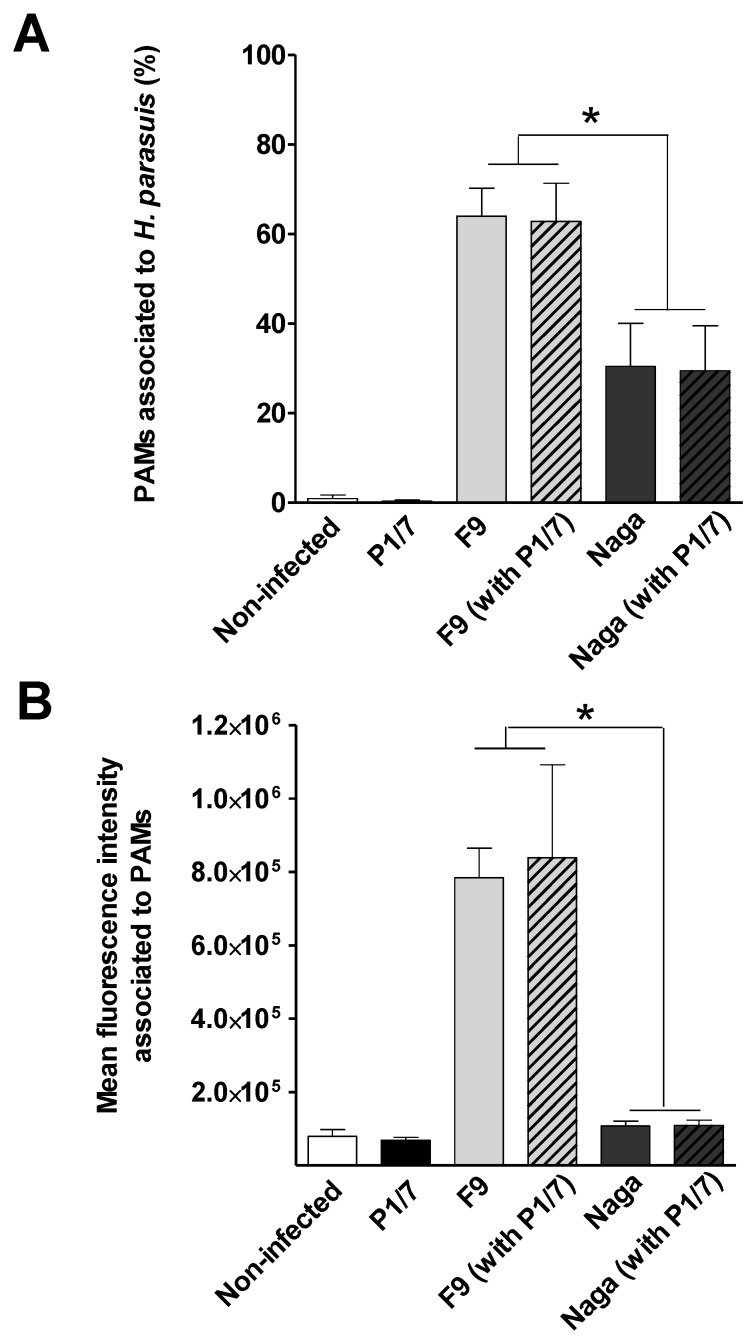
Association of *H. parasuis* to primary alveolar macrophages (PAMs) assessed by FACS. PAMs were incubated with a FITC-stained *H. parasuis* bacterial suspension at a MOI of 500 for 2 h. Cells were then washed and harvested in PBS with bovine serum albumin. The percentage of cells associated with *H. parasuis* (**A**) and the quantity of *H. parasuis* associated per cells, given by the mean fluorescence intensity (MFI) values (**B**), were determined by FACS. The effect of a pre-infection with *S. suis* on the association of *H. parasuis* to PAMs was evaluated through a pre-treatment of the cells with unstained *S. suis* P1/7 strain (MOI: 50) for 1 h. Mock infected and cells infected with unstained *S. suis* (P1/7) alone were used as negative controls. Data are expressed as means ± standard errors from at least four independent experiments. An asterisk indicates significant differences between samples (*p* < 0.05). Naga = Nagasaki.

It has been previously reported that low virulent strains of *H. parasuis* present higher levels of association with PAMs than virulent strains [16]. Accordingly, in the present study, the non-virulent F9 strain presented a significant higher level of association to PAMs than the Nagasaki strain, in terms of % of positive cells as well as in mean fluorescence intensity levels (MFI) (Figure 5). 

It has been proposed that the *S. suis* is able to destabilize the lipid rafts on the surface of macrophages which contain lactosylceramide, preventing the interaction and phagocytosis of encapsulated strains [10]. In this way, *S. suis* prevents phagocytosis and remains extracellular [3]. This effect would be attributed to the CPS, since the use of purified CPS was able to inhibit not only the phagocytosis of a non-encapsulated *S. suis* strain but also that of an heterologous species (Group B *Streptococcus*) [10]. To evaluate a possible role of *S. suis* on *H. parasuis* association to PAMs, cells were pre-infected with the high-virulent *S. suis* P1/7 strain followed by *H. parasuis* strains. Since the capsular polysaccharide of all *S. suis* serotype 2 strains are chemically and antigenically homogenous [32], strain P1/7 was chosen as a representative strain to test this hypothesis. Interestingly, a pre-infection with *S. suis* serotype 2 did not change the association levels of any of the *H. parasuis* strains (Figure 5), confirming that these two bacterial species use different receptors. 

**Figure 6:**
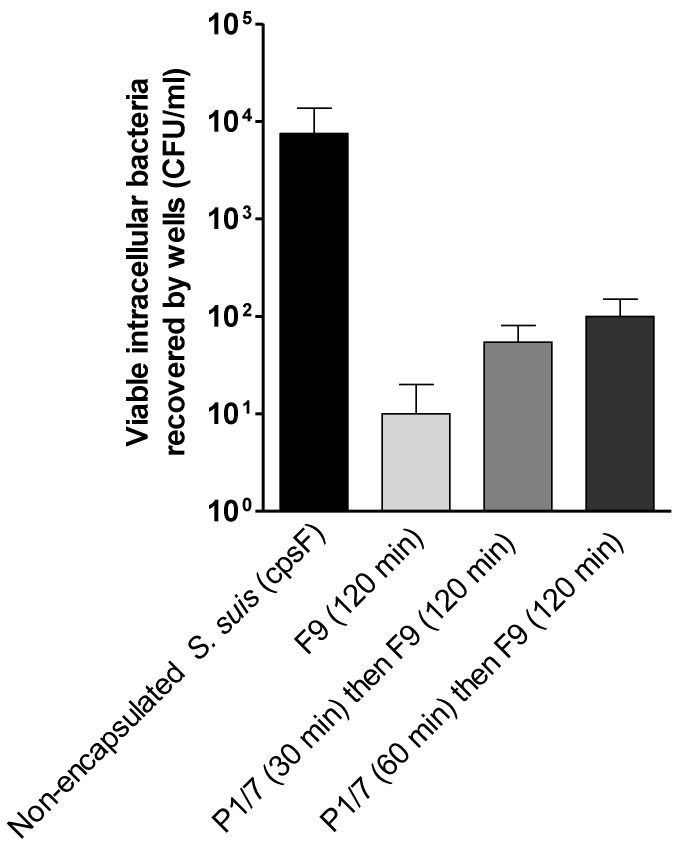
Phagocytosis of *H. parasuis* by primary alveolar macrophages (PAMs) as assessed by the antibiotic-protection assay. PAMs were incubated in presence of low-virulent *H. parasuis* F9 strain with a MOI: 100 for 120 min, washed and antibiotics were added to cell culture media and incubated for additional 120 min. PAMs were washed and lyzed in cold water to disrupt cell membranes in order to release intracellular bacteria. The suspension was plated on chocolate agar and colony forming units (CFU) were counted after an incubation time of 24–48 h. The effect of a pre-infection with *S suis* P1/7 strain on the phagocytosis of *H. parasuis* to PAMs was evaluated after a pre-treatment of cells with *S. suis* (MOI: 100) for 30 or 60 min. Cells infected with non-encapsulated *S. suis* were used as positive control to confirm the phagocytosis capacity of the cells. Data are expressed as means ± standard errors from at least three independent experiments.

As mentioned, in the lungs, low-virulent strains of *H. parasuis* can be eliminated through phagocytosis by macrophages [16]. *H. parasuis* is a ubiquitous bacterium in the upper respiratory tract of conventional pigs, which may be the reason that some strains from the upper respiratory tract may sometimes be found in the lungs. On the other hand, in some cases, strains with a low-virulent profile can be isolated from lungs of affected animals [18]. Since clinical cases of *H. parasuis* can be present in some farms as a consequence of co-infections (such as Porcine Reproductive and Respiratory Syndrome) [12], it is possible that under those circumstances low-virulent strains are able to induce disease. In the present study, only the low-virulent F9 strain could be recovered intracellularly in the antibiotic-protection assay with PAMs (Figure 6). Indeed, the high-virulent Nagasaki strain could be hardly found inside these cells and could not be recovered in most of the experiments (not shown). Similarly, very low levels of phagocytosis of this strain has previously been reported [16]. 

As mentioned above, the CPS of *S. suis* serotype 2 was described to be able to inhibit not only the phagocytosis of a non-encapsulated *S. suis* strain but also that of an heterologous species (Group B *Streptococcus*) [10]. We hypothesized that *S. suis* may prevent phagocytosis of low-virulent *H. parasuis* which would allow the latter to replicate extracellularly and increase the inflammatory reaction and causing disease. However, our results do not seem to support such hypothesis. A pre-treatment of the cells with encapsulated *S. suis* for 30 or 60 min did not affect the phagocytosis of the *H. parasuis* low-virulent F9 strain as shown by similar levels of bacteria recovered from the wells (Figure 6). It has been suggested that phagocytosis of *H. parasuis* is probably not dependent on a specific receptor, since phagocytosis of low-virulence strains was not affected by the presence high-virulent strains [16].

## 3. Materials and Methods 

### 3.1. Bacterial Strains

Two different strains of *S. suis* serotype 2 were used in this study (Table 1); the well characterized high-virulent strain P1/7 (ST1) from Europe and the intermediate-virulent strain 89-1591 (ST25) from Canada [33]. Bacteria were cultured as previously described with some modifications [34]. Briefly, *S. suis* strains were cultivated on Colombia sheep blood agar plates (Oxoid, Burlington, ON, Canada), which were incubated at 37 °C for 16 h with 5% CO_2_. For cell infections, 5 mL of Todd Hewitt Broth (THB; Difco, Mississauga, ON, Canada) was inoculated with a few colonies of *S. suis* and incubated for 16 h at 37 °C, with agitation. To obtain the final culture, 10 mL of fresh medium was inoculated with 400 μL of the overnight culture and incubated at 37 °C under agitation until reaching the exponential growth phase when an optical density 0.6 (OD_600nm_) was obtained. Two different strains of *H. parasuis* were also used. The virulent Nagasaki strain (originally isolated from a case of septicemia with meningitis in Japan) and low-virulent F9 strain, isolated from the nasal cavities of a pig in Spain [27] (Table 1). Bacteria were grown on chocolate agar plates (Oxoid, Burlington, ON, Canada) as previously described [16], incubated 16 h at 37 °C with 5% CO_2_ and harvested with sterile phosphate buffered saline (PBS). After centrifugation, bacteria (*S. suis* and *H. parasuis*) were suspended to the appropriate concentration in cell culture media without antibiotics. 

### 3.2. Cell Culture

The Newborn Pig Tracheal cells (NPTr) and primary alveolar macrophages (PAMs) were used for co-infection studies. NPTr cells were cultured as described before [11,24,39]. Briefly, cells were grown at 37 °C with 5% CO_2_ in Dulbelcco’s Minimum Essential Medium (DMEM; Gibco, Burlington, ON, Canada) supplemented with 10% (*v*/*v*) heat-inactivated fetal bovine serum (FBS; Gibco, Burlington, ON, Canada), penicillin-streptomycin (100 U/mL; Gibco, Burlington, ON, Canada) and gentamycin (0.04 mg/mL; Gibco, Burlington, ON, Canada). For assays, cells were treated with 0.1% trypsin in 0.03% ethylenediaminetetracetic acid (EDTA) solution (Gibco), suspended in fresh culture media and distributed into 24 wells tissue culture plates (Falcon, Mississauga, ON, Canada). The media was replaced after 24 h by cell culture medium without antibiotic and the cells were incubated until they reached confluence, with a final concentration of 1 × 10^5^ cells/mL. The day of the experiment, cells were washed three times with PBS and fresh cell culture medium without antibiotic was added to the wells. 

To harvest the PAMs, bronchoalveolar lavages were performed with sterile PBS on lungs from 5 six-week old piglets from a high-health status farm as previously described [40] but without the use of antibiotics. These studies were carried out in strict accordance with the recommendations of and approved by the University of Montreal Animal Welfare Committee guidelines and policies (protocol number Rech-1570). Cells were washed twice with DMEM and frozen in liquid nitrogen to a final concentration of 2 × 10^7^ cells/mL in DMEM supplemented with 20% (*v*/*v*) FBS use. For the experiments, PAMs were thawed in warm DMEM supplemented with 10% FBS, centrifuged at 800× *g* and suspended at a concentration of 1 × 10^5^ cells/mL in fresh culture medium. Five milliliters of the suspension were distributed in each well of 6-well tissue Primaria culture plates (Falcon, Mississauga, ON, Canada). PAMs were further incubated overnight at 37 °C with 5% CO_2_ in a humid atmosphere. Sterility controls were done in parallel.

### 3.3. Adhesion and Invasion of NPTr by S. suis and H. parasuis 

The so-called adhesion assay (which in fact quantifies total intracellular and surface-adherent bacteria) was performed as described before [19,24] with some modifications. Briefly, cells were infected with either *S. suis* or *H. parasuis* with a MOI of 5 by removing the cell culture media and by replacing it with 1 mL of 5 × 10^5^ of the bacterial suspension. This MOI was established as being optimal during standardization tests. Bacteria were cultured as previously reported [19,24]. The number of colony forming units (CFU)/mL in the final suspension before each experiment was determined by plating *S. suis* samples onto Todd-Hewitt agar (Difco, Mississauga, ON, Canada) or *H. parasuis* samples onto chocolate agar plates (Oxoid, Burlington, ON, Canada) using an Autoplate 4000 automated spiral plater (Spiral Biotech, Norwood, MA, USA). Cell culture plates were then infected with the bacterial suspensions and centrifuged at 800× *g* for 10 min in order to bring bacteria into close contact with cells [19] and further incubated at different incubation times (from 15 min up to 2 h) for adhesion assays. For simultaneous co-infections, 500 μL of both twice-concentrated bacterial suspensions (total of 1 mL; final MOI of 5 for each bacterial species) were added to the wells. For sequential co-infections, cells were pre-infected with one pathogen (either *S. suis* or *H. parasuis*) at a MOI of 5 (5 × 10^5^ bacteria), centrifuged at 800× *g* and incubated for 15 min at 37 °C with 5% CO_2_. In selected experiments and in order to saturate receptors, an MOI of 500 was also used. Cells were then washed 3 times with sterile PBS and 1 mL of the second bacterial suspension (MOI: 5, 5 × 10^5^ bacteria) was added to the wells. The plates were centrifuged again and further incubated up to 2 h. These conditions were established during standardization tests. 

For adhesion studies and after the incubation time, cells were washed five times with PBS and disrupted with sterile ice-cold deionized water followed by cell scraping from the bottom of the well in order to liberate cell-associated bacteria. The cell suspensions were plated and incubated at 37 °C for 24–48 h on THA plates (to count *S. suis* colonies only) or chocolate agar to count either *H. parasuis* or both bacterial species which could be easily differentiated by colony morphology. Levels of adhesion were expressed as the total number of CFU recovered per well. For the invasion assay, a method similar to that of the adhesion assay was followed, except that after 2 h of incubation, the NPTr cell monolayers were washed twice with PBS and 1 mL of cell culture medium containing 100 µg of gentamicin and 5 µg of penicillin G (Invitrogen, Burlington, ON, Canada) was added to each well. The plates were then further incubated for 1 h at 37 °C with 5% CO_2_ to kill extracellular and surface-adherent bacteria. Cells were washed three times and the last wash was plated to confirm antibiotic activity. Cells were then disrupted and bacterial CFU numbers were determined as described above. Levels of invasion were expressed as the total number of CFU recovered per well.

### 3.4. Induction of Pro-Inflammatory Cytokine Expression 

For cell activation, NPTr and PAMs were infected with either *S. suis* or *H. parasuis*, or with both bacterial species simultaneously. All manipulations were carried out on LPS-free conditions. Cell culture media was removed from the wells and replaced by 1000 μL of bacterial suspension (MOI: 10, 1 × 10^6^ bacteria) for single infections, or with 500 μL of both twice-concentrated bacterial suspensions for simultaneous co-infections. The plates were centrifuged 10 min at 800× *g* and incubated 6 h (NPTr) or 12 h (PAMs) at 37 °C with 5% CO_2_. MOI and incubation times were chosen based on cytotoxicity studies, in order to work under non-toxic conditions. The cells were washed twice with warm PBS and homogenized in 1000 μL of QIAzol (Qiagen, Toronto, ON, Canada). The samples were frozen at −80 °C until RNA extraction. 

### 3.5. RNA Extraction, cDNA Construct and RT-qPCR

The RNA extractions using chloroform were performed according to kit instructions (QIAzol, Qiagen, Toronto, ON, Canada). Purified RNA was suspended in 20 μL of DNAse and RNAse-free water (Fisher, Ottawa, ON, Canada) and was quantified with NanoDrop 1000 (Fisher, Ottawa, ON, Canada). Complementary DNA (cDNA) was synthesized from 500 ng of sample RNA with QuantiTect Reverse Transcription Kit (Qiagen, Toronto, ON, Canada) according to the manufacturer’s instructions. The cDNA was diluted seven times in water for qPCR analysis. 

Primers (IDT, Coralville, IA, USA) used for quantitative PCR are listed in Table 2. A CFX96 rapid thermal cycler system (Bio-Rad, Mississauga, ON, Canada) showed that primers had a PCR amplification efficiency ranking between 90 and 110%. The cDNA was amplified by qPCR as described by Lecours et al. [41]. Briefly, the cDNA was amplified using SsoFast EvaGreen Supermix kit (Bio-Rad, Mississauga, ON, Canada). The PCR amplification program for all cDNA consisted of an enzyme activation step of three min at 98 °C, followed by 40 cycles of denaturation for 2 s at 98 °C and an annealing/extension step for 5 s at 58 °C. Two genes, PPIA and Hypox, were used as the normalizing genes to compensate for potential differences in cDNA amounts [11]. Fold changes in gene expression were calculated using the normalized gene expression (∆∆Cq) calculation method of the CFX software manager (v.2.1: Bio-Rad, Mississauga, ON, Canada). The non-infected cells group was used as the calibrator reference in the analysis. The results originated from at least three independent experiments.

### 3.6. Cytotoxicity

The cell supernatants from infected cells under different assay conditions were used for LDH detection using the Cyto Tox 96 Non-Radioactive Cytotoxicity assay kit (Promega, Madison, WI, USA) according to manufacturer’s instructions. Medium from non-infected cells and supernatant from non-infected cells lyzed in pure cold water were used as negative and positive controls, respectively. 

### 3.7. Effect of S. suis on H. parasuis Association to and Phagocytosis by PAMs 

#### 3.7.1. Association of *H. parasuis* to PAMs by FACS

In order to evaluate *H. parasuis* association to PAMs by flow cytometry (FACS), bacteria were first stained. Five milliliters of a *H. parasuis* bacterial suspension of an OD_600nm_ of 1.0 were incubated with 2 ng/mL of fluorescein isothiocyanate (FITC; Sigma-Aldrich, Oakville, ON, Canada) for 45 min at 37 °C with agitation [16]. Bacteria were washed three times with PBS supplemented with 1% (*v*/*v*) of FBS. Tenfold dilutions were plated on chocolate agar to determine the final concentration of bacteria. Then, PAMs were infected with 5 mL of stained *H. parasuis* (MOI: 500, which corresponds to 5 × 10^5^ bacteria) suspension. Plates were incubated for 2 h at 37 °C, followed by two washes with warm PBS and cells were harvested with a scraper in PBS supplemented with 1% (*w*/*v*) bovine serum albumin (Difco, Mississauga, ON, Canada) [16]. The effect of a pre-infection with *S. suis* on the association of *H. parasuis* to PAMs was evaluated by a pre-treatment of the cells with virulent or intermediate virulent *S. suis* strains (MOI: 50 which corresponds to 5 × 10^6^ bacteria) by replacing the cell culture media with 5 mL of the unstained *S. suis* suspension and by incubating the plates at 37 °C for 1 h, followed by FITC-stained *H. parasuis* infection as described above. Mock-infected and cells infected with unstained *S. suis* only were used as control. Different MOIs were evaluated in pre-standardization tests in order to select the above indicated experimental conditions.

#### 3.7.2. Phagocytosis of *H. parasuis* by PAMs by the Antibiotic-Protection Assay 

The viable intracellular count of *H. parasuis* was also assessed to determine the effect of *S. suis* on its phagocytosis by PAMs. Cell culture media was replaced by a suspension of *S. suis* ST1 strain P1/7 (MOI: 100) and the plates were centrifuged at 800× *g* for 10 min. Non-infected cells were included as controls. Cells were then further incubated 30 or 60 min and cell culture medium was replaced with a suspension of *H. parasuis* (MOI: 100, 1 × 10^7^ bacteria). Plates were centrifuged and phagocytosis was left to proceed for 2 h, to allow optimal phagocytosis, determined during preliminary studies with different incubation times and MOIs. After incubation, cell monolayers were washed twice with PBS and incubated 2 h with medium containing antibiotics to kill extracellular bacteria, as described above. Supernatant controls were taken during every test to confirm the activity of the antibiotics. After antibiotic treatment, cell monolayers were washed three times with PBS, lysed with water and vigorous pipetting and viable intracellular bacteria determined by plating appropriate dilutions as described above. Each test was repeated at least three times (with cells from 3 different animals) in independent experiments and the number of CFU/mL was determined as described above. To confirm the phagocytosis activity of the cells, a non-encapsulated *S. suis* mutant was used as positive control [41] (Table 1). Lyzed cells and internalized bacteria were plated on chocolate agar (Oxoid, Burlington, ON, Canada) and incubated for 24–48 h at 37 °C. A bacterial count was performed to determine the rate of *H. parasuis* phagocyted by PAMs. 

### 3.8. Statistical Analysis 

All data are expressed as means ± standard errors. SigmaPlot Software (v.11.0) was used for data analysis. Significance was determined with one-way analysis of variance (ANOVA) or Student’s unpaired t test where appropriate depending on the experiment. *p* values of <0.05 were considered significant. Results reflect mean values from at least three independent experiments. 

## 4. Conclusions

This study showed that a low-virulence strain of *H. parasuis* adheres to swine respiratory epithelial cells and PAMs at higher levels than a virulent strain as well as both *S. suis* strains. In addition, *H. parasuis* induce a significant higher level of pro-inflammatory cytokines than *S. suis* from both cell types. Within *H. parasuis* strains, the low-virulent F9 strain induces in general higher levels of pro-inflammatory cytokines than the virulent Nagasaki strain. Finally, and under the conditions used in this study, it has been shown that, overall, *S. suis* and *H. parasuis* have limited in vitro interactions between them, regardless to their level of virulence. Although it has been previously described that the CPS of *S. suis* serotype 2 has anti-phagocytic properties against an heterologous bacterial species, pre-treatment of PAMs did not have a clear effect on the phagocytosis of a low-virulence strain of *H. parasuis*. Although not clear interactions could be observed between the two bacterial species in the present study, further in vitro experiments (such as biofilm formation), a higher number of tested strains as well as in vivo studies should be carried out to reach more definitive conclusions. 

## Figures and Tables

**Table 1:** *Streptococcus suis* serotype 2 and *Haemophilus parasuis* strains used in this study.

Strain	ST or Serovar	Country	Host	Virulence/Sly	Reference
*S. suis* serotype 2					
P1/7	1	UK	Pig	High virulent/Yes	[35]
89-1591	25	Canada	Pig	Intermediate virulent/No	[36,37]
*H. parasuis*					
F9	6	Spain	Pig	Low-virulent	[27]
Nagasaki	5	Japan	Pig	Virulent	[38]

ST: sequence type as describe by multilocus sequence typing; Sly: presence of suilysin.

**Table 2:** Porcine-specific primer sequences used in the study for pro-inflammatory cytokine detection by real-time quantitative RT-qPCR.

Gene Name	Forward	Reverse
**IL-6**	ACTCCCTCTCCACAAGCGCCTT	TGGCATCTTCTTCCAGGCGTCCC
**IL-8**	TGTGAGGCTGCAGTTCTGGCAAG	GGGTGGAAAGGTGTGGAATGCGT
**Hypox**	GCAGCCCCAGCGTCGTGATT	CGAGCAAGCCGTTCAGTCCTGT
**PPIA**	TGCAGACAAAGTTCCAAAGACAG	GCCACCAGTGCCATTATGG

Hypox: hypoxanthine phosphoribosyl transferase 1; PPIA: Peptidylprolyl isomerase A.

## Abbreviations

The following abbreviations are used in this manuscript:

cDNA	Complementary DNA
CFU	Colony forming unit
CPS	Capsular polysaccharide
DMEM	Dulbecco’s modified Eagle’s Medium
EDTA	Ethylenediaminetetraacetic acid
FACS	Fluorescence associated cell sorting
FBS	Fetal bovine serum
FITC	Fluoroscein isothiocyanate
GbO3	globotriaosylceramide
Hypox	hypoxanthine phosphoribosyl transferase 1
IL	Interleukin
LDH	lactate dehydrogenase
LPS	Lipopolysaccharide
MFI	Mean fluorescence intensities
MOI	Multiplicity of infection
NPTr	Newborn Pig Tracheal cells
PAMs	Primary alveolar macrophages
PBS	Phosphate-buffered saline
PCR	Polymerization chain reaction
PPIA	Peptidylprolyl isomerase A
RT-PCR	Reverse transcription polymerization chain reaction
ST	Sequence type
THA	Todd-Hewitt broth agar
THB	Todd-Hewitt broth
VtaA	Trimeric autotransporter

## References

[1] Gottschalk M., Segura M., Xu J. (2007). *Streptococcus suis* infections in humans: The chinese experience and the situation in North America. Anim. Health Res. Rev..

[2] Costa-Hurtado M., Aragon V. (2013). Advances in the quest for virulence factors of *Haemophilus parasuis*. Vet. J..

[3] Fittipaldi N., Segura M., Grenier D., Gottschalk M. (2012). Virulence factors involved in the pathogenesis of the infection caused by the swine pathogen and zoonotic agent *Streptococcus suis*. Future Microbiol..

[4] Okura M., Osaki M., Nomoto R., Arai S., Osawa R., Sekizaki T., Takamatsu D. (2016). Current taxonomical situation of *Streptococcus suis*. Pathogens.

[5] Zheng H., Qiu X., Roy D., Segura M., Du P., Xu J., Gottschalk M. (2017). Genotyping and investigating capsular polysaccharide synthesis gene loci of non-serotypeable *Streptococcus suis* isolated from diseased pigs in Canada. Vet. Res..

[6] Goyette-Desjardins G., Auger J.P., Xu J., Segura M., Gottschalk M. (2014). *Streptococcus suis*, an important pig pathogen and emerging zoonotic agent-an update on the worldwide distribution based on serotyping and sequence typing. Emerg. Microbes Infect..

[7] Fittipaldi N., Xu J., Lacouture S., Tharavichitkul P., Osaki M., Sekizaki T., Takamatsu D., Gottschalk M. (2011). Lineage and virulence of *Streptococcus suis* serotype 2 isolates from North America. Emerg. Infect. Dis..

[8] Segura M., Calzas C., Grenier D., Gottschalk M. (2016). Initial steps of the pathogenesis of the infection caused by *Streptococcus suis*: Fighting against nonspecific defenses. FEBS Lett..

[9] Segura M., Fittipaldi N., Calzas C., Gottschalk M. (2017). Critical streptococcus suis virulence factors: Are they all really critical?. Trends Microbiol..

[10] Houde M., Gottschalk M., Gagnon F., Van Calsteren M.R., Segura M. (2012). *Streptococcus suis* capsular polysaccharide inhibits phagocytosis through destabilization of lipid microdomains and prevents lactosylceramide-dependent recognition. Infect. Immun..

[11] Dang Y., Lachance C., Wang Y., Gagnon C.A., Savard C., Segura M., Grenier D., Gottschalk M. (2014). Transcriptional approach to study porcine tracheal epithelial cells individually or dually infected with Swine Influenza virus and *Streptococcus suis*. BMC Vet. Res..

[12] Oliveira S., Pijoan C. (2004). *Haemophilus parasuis*: New trends on diagnosis, epidemiology and control. Vet. Microbiol..

[13] Ma L., Wang L., Chu Y., Li X., Cui Y., Chen S., Zhou J., Li C., Lu Z., Liu J. (2016). Characterization of chinese *Haemophilus parasuis* isolates by traditional serotyping and molecular serotyping methods. PLoS ONE.

[14] Lacouture S., Rodriguez E., Strutzberg-Minder E., Gottschalk M. (2017). Serotyping of *Haemophilus parasuis* field isolates from diseased pigs in quebec by indirect hemagglutination assay and multiplex polymerase chain reaction (pcr). Can. Vet. J..

[15] Frandoloso R., Martinez-Martinez S., Gutierrez-Martin C.B., Rodriguez-Ferri E.F. (2012). *Haemophilus parasuis* serovar 5 nagasaki strain adheres and invades pk-15 cells. Vet. Microbiol..

[16] Olvera A., Ballester M., Nofrarias M., Sibila M., Aragon V. (2009). Differences in phagocytosis susceptibility in *Haemophilus parasuis* strains. Vet. Res..

[17] Boerlin P., Poljak Z., Gallant J., Chalmers G., Nicholson V., Soltes G.A., MacInnes J.I. (2013). Genetic diversity of *Haemophilus parasuis* from sick and healthy pigs. Vet. Microbiol..

[18] Galofre-Mila N., Correa-Fiz F., Lacouture S., Gottschalk M., Strutzberg-Minder K., Bensaid A., Pina-Pedrero S., Aragon V. (2017). A robust pcr for the differentiation of potential virulent strains of *Haemophilus parasuis*. BMC Vet. Res..

[19] Wang Y., Gagnon C.A., Savard C., Music N., Srednik M., Segura M., Lachance C., Bellehumeur C., Gottschalk M. (2013). Capsular sialic acid of *Streptococcus suis* serotype 2 binds to swine influenza virus and enhances bacterial interactions with virus-infected tracheal epithelial cells. Infect. Immun..

[20] Ferrando M.L., Fuentes S., de Greeff A., Smith H., Wells J.M. (2010). Apua, a multifunctional alpha-glucan-degrading enzyme of *Streptococcus suis*, mediates adhesion to porcine epithelium and mucus. Microbiology.

[21] Wu N.H., Meng F., Seitz M., Valentin-Weigand P., Herrler G. (2015). Sialic acid-dependent interactions between influenza viruses and *Streptococcus suis* affect the infection of porcine tracheal cells. J. Gen. Virol..

[22] Tenenbaum T., Asmat T., Seitz M., Schroten H., Schwerk C. (2016). Biological activities of suilysin: Role in *Streptococcus suis* pathogenesis. Future Microbiol..

[23] Seitz M., Baums C.G., Neis C., Benga L., Fulde M., Rohde M., Goethe R., Valentin-Weigand P. (2013). Subcytolytic effects of suilysin on interaction of *Streptococcus suis* with epithelial cells. Vet. Microbiol..

[24] Bouchet B., Vanier G., Jacques M., Auger E., Gottschalk M. (2009). Studies on the interactions of *Haemophilus parasuis* with porcine epithelial tracheal cells: Limited role of los in apoptosis and pro-inflammatory cytokine release. Microb. Pathog..

[25] Auger E., Deslandes V., Ramjeet M., Contreras I., Nash J.H., Harel J., Gottschalk M., Olivier M., Jacques M. (2009). Host-pathogen interactions of *Actinobacillus pleuropneumoniae* with porcine lung and tracheal epithelial cells. Infect. Immun..

[26] De Greeff A., Benga L., Wichgers Schreur P.J., Valentin-Weigand P., Rebel J.M., Smith H.E. (2010). Involvement of nf-kappab and map-kinases in the transcriptional response of alveolar macrophages to *Streptococcus suis*. Vet. Microbiol..

[27] Costa-Hurtado M., Olvera A., Martinez-Moliner V., Galofre-Mila N., Martinez P., Dominguez J., Aragon V. (2013). Changes in macrophage phenotype after infection of pigs with *Haemophilus parasuis* strains with different levels of virulence. Infect. Immun..

[28] Fu S., Yuan F., Zhang M., Tan C., Chen H., Bei W. (2012). Cloning, expression and characterization of a cell wall surface protein, 6-phosphogluconate dehydrogenase, of *Haemophilus parasuis*. Res. Vet. Sci..

[29] Chen Y., Liu T., Langford P., Hua K., Zhou S., Zhai Y., Xiao H., Luo R., Bi D., Jin H. (2015). *Haemophilus parasuis* induces activation of nf-kappab and map kinase signaling pathways mediated by toll-like receptors. Mol. Immunol..

[30] Lecours M.P., Segura M., Fittipaldi N., Rivest S., Gottschalk M. (2012). Immune receptors involved in *Streptococcus suis* recognition by dendritic cells. PLoS ONE.

[31] Chen Y., Zhou S., Hua K., Xiao H., Li Z., Liu M., Luo R., Bi D., Zhou R., Jin H. (2015). *Haemophilus parasuis* infection activates chemokine rantes in pk-15 cells. Mol. Immunol..

[32] Van Calsteren M.R., Gagnon F., Lacouture S., Fittipaldi N., Gottschalk M. (2010). Structure determination of *Streptococcus suis* serotype 2 capsular polysaccharide. Biochem. Cell Biol..

[33] Auger J.P., Fittipaldi N., Benoit-Biancamano M.O., Segura M., Gottschalk M. (2016). Virulence studies of different sequence types and geographical origins of *Streptococcus suis* serotype 2 in a mouse model of infection. Pathogens.

[34] Vanier G., Segura M., Friedl P., Lacouture S., Gottschalk M. (2004). Invasion of porcine brain microvascular endothelial cells by *Streptococcus suis* serotype 2. Infect. Immun..

[35] Auger J.P., Christodoulides M., Segura M., Xu J., Gottschalk M. (2015). Interactions of *Streptococcus suis* serotype 2 with human meningeal cells and astrocytes. BMC Res. Notes.

[36] Athey T.B., Teatero S., Takamatsu D., Wasserscheid J., Dewar K., Gottschalk M., Fittipaldi N. (2016). Population structure and antimicrobial resistance profiles of *Streptococcus suis* serotype 2 sequence type 25 strains. PLoS ONE.

[37] Holden M.T., Hauser H., Sanders M., Ngo T.H., Cherevach I., Cronin A., Goodhead I., Mungall K., Quail M.A., Price C. (2009). Rapid evolution of virulence and drug resistance in the emerging zoonotic pathogen *Streptococcus suis*. PLoS ONE.

[38] Morozumi T., Nicolet J. (1986). Some antigenic properties of *Haemophilus parasuis* and a proposal for serological classification. J. Clin. Microbiol..

[39] Ferrari M., Scalvini A., Losio M.N., Corradi A., Soncini M., Bignotti E., Milanesi E., Ajmone-Marsan P., Barlati S., Bellotti D. (2003). Establishment and characterization of two new pig cell lines for use in virological diagnostic laboratories. J. Virol. Methods.

[40] McCaig W.D., Loving C.L., Hughes H.R., Brockmeier S.L. (2016). Characterization and vaccine potential of outer membrane vesicles produced by *Haemophilus parasuis*. PLoS ONE.

[41] Lecours M.P., Segura M., Lachance C., Mussa T., Surprenant C., Montoya M., Gottschalk M. (2011). Characterization of porcine dendritic cell response to *Streptococcus suis*. Vet. Res..

